# Trajectories of Work Absence in England due to a Musculoskeletal or Mental Health Condition: An Electronic Health Record Cohort Study

**DOI:** 10.1007/s10926-025-10342-y

**Published:** 2025-11-28

**Authors:** Amardeep Legha, James Bailey, Victoria K. Welsh, Kelvin P. Jordan, Clare Holdsworth, Gwenllian Wynne-Jones

**Affiliations:** 1 Faculty of Medicine and Health Sciences, and Versus Arthritis/Medical Research Council Centre for Musculoskeletal Health and Work, School of Medicine, Keele University, Keele, Staffordshire, UK; 2 Department of Applied Health Sciences, School of Health Sciences, College of Medicine and Health, University of Birmingham, Birmingham, UK; 3 National Institute for Health and Care Research (NIHR) Birmingham Biomedical Research Centre, Birmingham, UK; 4 Primary Care Centre Versus Arthritis, School of Medicine, Keele University, Keele, Staffordshire, UK; 5 School of Geography, Geology and the Environment, Keele University, Keele, Staffordshire, UK

**Keywords:** Sickness absence, Fit notes, Musculoskeletal conditions, Mental health conditions, Trajectories, Latent class analysis

## Abstract

**Purpose:**

To derive common patterns (trajectories) of work absence over time due to a musculoskeletal (MSK) or mental health (MH) condition in an English population and determine associations of these absence trajectories with health and sociodemographic characteristics.

**Methods:**

This retrospective cohort study used primary care data for 43,130 and 62,355 economically active individuals with an incident work absence (as measured by receipt of fit notes) due to a MSK or MH condition, respectively, between 2016 and 2018. Latent class growth analysis was used to define trajectories (through issuance of fit notes), and trajectory-covariate association analysis was performed through multivariable multinomial logistic regression.

**Results:**

Five common trajectories of work absence associated with MSK and MH conditions were determined over a one-year follow-up. The two most common trajectories consisted of low absence (a ‘Single’ fit note and ‘Short Term’ absence), while the two least common trajectories were characterized by longer-term absence of six months or more (‘Chronic Sustained’ and ‘Chronic Fast Decreasing’), and the fifth by intermittent absence. Individuals associated with the two longer-term absence trajectories were older, living in the North or Midlands or most deprived areas of England, prescribed opioids, and current smokers.

**Conclusions:**

This study has highlighted different patterns of sickness absence due to a MSK or MH condition and profiles of individuals associated with longer-term absence. Earlier and more targeted health and work intervention toward these high-risk subgroups, alongside policy interventions to reduce health inequalities, could help alleviate the rising rate of long-term sickness absence and economic inactivity.

**Supplementary Information:**

The online version contains supplementary material available at https://doi.org/10.1007/s10926-025-10342-y.

## Introduction

Long-term sickness absence has been rising in the UK. Between spring 2019 and summer 2022, the number of working-age adults that were economically inactive (not working nor seeking employment) due to long-term sickness absence increased by half a million [1]. Being in work is generally beneficial for physical and mental health, while being off work is associated with poorer health and well-being [2]. While the majority of people with a sickness absence tend to return-to-work (RTW) quickly, approximately 10% go on to have longer-term absences of > 12 months [3]. Once an individual is on a long-term absence, it becomes progressively harder to RTW, and more adverse health and social problems are experienced [2].

In the UK, if an individual is off work for more than seven consecutive days due to a medical problem, a ‘fit note’ is issued from a healthcare professional that details the medical advice the individual has received regarding their fitness to work and can be used to claim statutory sick pay through the state [4, 5]. Fit notes are recorded in primary care as electronic health records. The most common reason for fit note issuance in England is due to a mental health (MH) condition, followed by a musculoskeletal (MSK) condition (with 37.6% and 17.5% of all fit notes issued between April 2021 and March 2024 due to MH and MSK conditions, respectively) [6].

Many existing studies of individuals with a sickness absence involve use of a dichotomous absence measure (absent/not absent) and analysis through either a cross-sectional approach based on a single-time point (using methods such as logistic regression), or a time-to-event approach (such as Cox regression) [7, 8]. However, RTW is a complex and dynamic process that changes with time, and both the cross-sectional and time-to-event approaches may be sub-optimal in capturing this as they treat RTW as a fixed status. In contrast, trajectory analysis is an approach that allows for repeated measures of absence data to be used to assign individuals into common subgroups (trajectories) based on duration and number of RTW spells. During an initial consultation with a healthcare professional for sickness absence, it is challenging to determine which patients are at the highest risk of a sustained long-term work absence; determining common trajectories of work absence and the patient characteristics associated with them may assist with this.

### Aims

This study has two main aims:

1. To derive common longitudinal trajectories of work absence as measured by receipt of fit notes, for a population consulting their healthcare professional with a MSK or MH condition
2. To identify health and sociodemographic characteristics associated with longer-term absence trajectories

## Methods

### Defining the Study Population

The study used data from a large UK primary care database: the Clinical Practice Research Datalink (CPRD) Aurum [9], and the study was approved by the CPRD Research Governance (protocol reference number 21_000665 made available to reviewers). The February 2022 release of CPRD Aurum (https://doi.org/10.48329/gcgx-f815) [10] was used, which contained over 13.4 million individuals that were currently registered in UK practices (this corresponded to 19.9% of the UK population). CPRD Aurum has been shown to be representative of the general English population in terms of age and gender, as well as deprivation and geographical spread (comparing a mid-2017 snapshot of CPRD Aurum data to mid-2017 data on the broader English population from the Office for National Statistics) [9].

The study population was based in English practices, aged 16–66 at time of a first ever recorded fit note (defined through searching a person’s lifetime history of fit notes recorded in the CPRD Aurum database) due to either a MSK or MH condition between 2016 and 2018, and had at least two years prior registration at their practice. As reason for fit note data is not available in CPRD, an assumption was made that the fit note could be attributed to a MSK or MH condition if there was a recorded MSK or MH consultation, on the day of, or in the two weeks prior to first ever fit note issuance.

MSK and MH conditions were defined using Read/SNOMED codes developed in a previous CPRD study [11]. MSK conditions were osteoarthritis, inflammatory MSK, and the most common regional pain (back pain, knee pain, hip pain, hand/wrist pain). MH conditions were depression, anxiety, and stress. These code lists are publicly available on the Keele Research Repository (https://doi.org/10.21252/878s-x990).

Included patients were censored at earliest of three years of follow-up after index fit note date, age ≥ 67 years (defined using year of birth and subtracting from year of follow-up), death, de-registration, and last collection date.

### Defining Trajectory Follow-Up and Interval Lengths

As fit note duration data were largely not available in the CPRD Aurum database, the repeated fit note measure used to assess patterns of absence was a binary yes/no for a recorded fit note issuance (coded as 1/0) in each given time interval during follow-up. The initial MSK or MH condition incident fit note was excluded from the trajectory definition, as all individuals received this.

Five different approaches to follow-up and interval length were used in this study to identify the optimal method for analysis (Table 1). A minimum of two-monthly time interval lengths were chosen to avoid underestimating recurrent fit note issuance (around 50% of all fit notes issued in England due to a MSK or MH condition in England have a duration of more than one month [12]; therefore, choosing monthly intervals would be too short).

**Table 1:** The five interval approaches used for trajectory derivation in this study

Approach	Follow-up length	Year one time interval length	Years two–three time interval length
1 (Short-Term)	One Year	Three-Monthly	N/A
2 (Short-Term)	One Year	Two-Monthly	N/A
3 (Long-Term)	Three Years	Six-Monthly	Six-Monthly
4 (Long-Term)	Three Years	Three-Monthly	Six-Monthly
5 (Long-Term)	Three Years	Two-Monthly	Six-Monthly

*N/A* not applicable

### Model Building Strategy

The trajectory models considered in this study were based on Latent Class Growth Analysis (LCGA). LCGA allows two or more trajectories ‘classes’ to be fitted to a study population. The probability of belonging to a trajectory class (or cluster), known as posterior probability, is estimated for each individual using the observed data, and the individual is assigned to the class that they have the greatest probability of belonging to. LCGA assumes that individuals follow the trajectory class that they are assigned to.

The model building strategy began with interval approach 1 (Table 1) for the index MSK condition cohort (follow-up of 1 year broken down into 4 three-monthly intervals), and initially with a two-class LCGA model. The number of classes in succeeding LCGA models was then progressively increased by one.

The following criteria were used to indicate better performing models:

- Akaike Information Criterion (AIC) [13] and Bayesian Information Criterion (BIC) [14] (lower values indicating better model fit);
- Statistically significant likelihood ratio tests (both the Lo-Mendell-Rubin [15] and the bootstrapped [16] likelihood ratio tests were used)—this indicates if a (*k* class) LCGA model fits the data better than a corresponding LCGA model with one fewer class (i.e., *k*-1 classes).
- Entropy and average posterior probabilities ≥ 0.7 (indicating better trajectory class separability)
- Minimum trajectory class prevalence ≥ 1% (to reduce the possibility of uncovering spurious trajectory classes)

Graphical assessment of observed compared to predicted probabilities of fit note issuance within trajectories was also performed to assess model fit, and trajectories were evaluated for interpretability and clinical meaningfulness. Finally, when comparing two competing models with *k* and *k* + 1 classes, if all of the criteria mentioned thus far were similar, the more parsimonious model (i.e., with fewer trajectory classes) was selected.

All LCGAs were fitted with a linear functional form.

Next, this strategy was repeated for individuals with an index MSK fit note for the four remaining approaches (Table 1). This process was repeated for individuals with a first ever fit note due to a MH condition.

For the two selected optimal LCGA trajectory models, a summary of the observed patterns of fit note issuance for individuals within a trajectory class was derived to assess individual variability of the final trajectory classes.

The reporting in this study was carried out in line with the Guidelines for Reporting on Latent Trajectory Studies (GRoLTS) checklist [17]. Stata MP version 17.0 (StataCorp, College Station, TX, USA) and Mplus Version 8.9 (Muthén & Muthén, Los Angeles, CA, USA) were used for the analyses in this study. In an effort to limit convergence issues, all trajectory analyses involved use of 500 random sets of starting values with 100 iterations [18].

Any absence measurement data that were missing due to censoring before end of follow-up were assumed to be Missing At Random, and Full Information Maximum Likelihood estimation was used to incorporate the non-missing repeated absence measurement data of such individuals (i.e., individuals were retained in the analysis as long as they had some non-missing data). A complete case sensitivity analysis to retain only individuals with complete follow-up data was also performed and did not change findings (results omitted for brevity).

### Characteristics Tested for Association with Trajectories

In total, 14 characteristics (see Table 2 for definitions and summaries) were included in the association analysis with the derived optimal absence trajectories:

- Sociodemographic Characteristics (4 variables)
- Health Characteristics (4 variables)
- Types of Treatment Received (4 variables)
- Comorbidity (2 variables)

**Table 2:** Summary of the *n* = 14 characteristics explored in this study

			Cohort	
Type of characteristic	Characteristic	Category definitions	MSK index fit note	MH index fit note
Sociodemographic	Sex	Female, Male	✔	✔
	Age	16–25, 26–35, 36–45, 46–55, 56–65 (years)	✔	✔
	Region^a^	North of England, Middle of England, South of England	✔	✔
	IMD^b^	1–5, Missing	✔	✔
Health	Number of MSK Consultations - Prior 2 Years^c^	0, 1, 2, ≥ 3	✔	✖
	Number of MH Consultations - Prior 2 Years^d^	0, 1, 2, ≥ 3	✖	✔
	Baseline MSK Condition	Back pain, Knee pain, Hand/Wrist pain, Hip pain, Inflammatory MSK, Osteoarthritis	✔	✖
	Baseline MH Condition	Stress, Anxiety, Depression, Anxiety and Depression	✖	✔
	Smoking Status^e^	Never, Current, Ex Smoker, Not Recorded	✔	✔
	BMI^f^	Underweight/Normal, Overweight, Obese, Not Recorded	✔	✔
Types of treatment received^g^	Opioids	Yes, No	✔	✔
	NSAIDs	Yes, No	✔	✔
	Gabapentinoids	Yes, No	✔	✔
	Antidepressants	Yes, No	✔	✔
Comorbidity	Polypharmacy^h^	0, 1–4, 5–9, ≥ 10	✔	✔
	CCI Score^g^	0, 1, ≥ 2	✖	✔
	Modified CCI Score^i^	0, 1, ≥ 2	✔	✖

*IMD* index of multiple deprivation, *MSK* musculoskeletal, *MH* mental health, *NSAIDs* non-steroidal anti-inflammatory drugs, *BMI* body mass index, *CCI* Charlson comorbidity index

^a^This is defined as the region of the General Practitioner Practice (primary care clinic) of the patient. North of England is defined as follows: North East, North West, Yorkshire and the Humber; Middle of England: East Midlands, West Midlands, East of England; and South of England: South East, South West, London

^b^Quintiles are used for IMD (1–5), where a higher score represents more deprived areas. An IMD of 5 represents the most deprived areas of England, and an IMD of 1 the least deprived areas

^c^Defined as the number of MSK consultations in the two years prior to index fit note date (excluding the index MSK consultation)

^d^Defined as the number of MH consultations in the two years prior to index fit note date (excluding the index MH consultation)

^e^In the five years prior to index fit note date and latest data retained

^f^Underweight/Normal: 10 <  = BMI < 25; Overweight: 25 <  = BMI < 30; Obese: 30 <  = BMI < 80; Not Recorded: no BMI data available or BMI < 10 or BMI >  = 80. BMI data were searched for in the five years prior to index fit note date and latest data retained

^g^In the two years prior to index fit note date

^h^Defined as a drug count (excluding Opioids, NSAIDs, Gabapentinoids, Antidepressants) in the two years prior to index fit note date

^i^In the two years prior to index fit note date, excluding Rheumatic Disease

All of these characteristics were accessed directly from the CPRD Aurum database, except Index of Multiple Deprivation (IMD) [19] which was accessed through a data-linkage. The latest version of patient-level linked IMD data was used (the 2019 version) and in quintile format; this is a relative measure, rather than absolute, that compares levels of deprivation of English neighborhoods at a lower-layer super output area level.

### Trajectory-Covariate Association Analyses

**S**eparately, for the index MSK and MH condition fit note cohorts, multivariable multinomial logistic regression was used to test for association between the sociodemographic and health characteristics with the optimal trajectories identified. The least severe absence trajectory class was used as the reference.

To preserve the original optimal trajectory classes, a three-step approach was used for this analysis, as developed by Vermunt (2010) [20]. This was achieved by first estimating the original optimal derived trajectory classes (step 1, as performed earlier in Methods Section), then adding relevant covariate data (step 2), and finally performing the multinomial logistic regression analysis by accounting for the uncertainty in class membership through specifying uncertainty rates associated with the derived classes (step 3). Adjusted odds ratios (ORs) of association with 95% confidence intervals (CIs) are presented.

### Patient and Public Involvement

A patient and public involvement and engagement meeting was held to disseminate the findings of this study to members of the public with lived experience of work absence and to seek their feedback on the interpretation of our study results.

## Results

There were 43,130 people included in this study with an initial MSK condition fit note (45.5% female), and 62,355 people with an initial MH condition fit note (59.2% female). Descriptive statistics are presented in Supplementary Materials S1.

### Optimal Trajectories of Work Absence

The optimal trajectory models chosen in this study were the same for both the index MSK and MH condition fit note cohorts: a five-class LCGA, under interval approach 2 (based on two-monthly recurring intervals of a one-year follow-up post-index fit note date). The process to choose the optimal models is explained in Supplementary Materials S2.

**Fig. 1:**
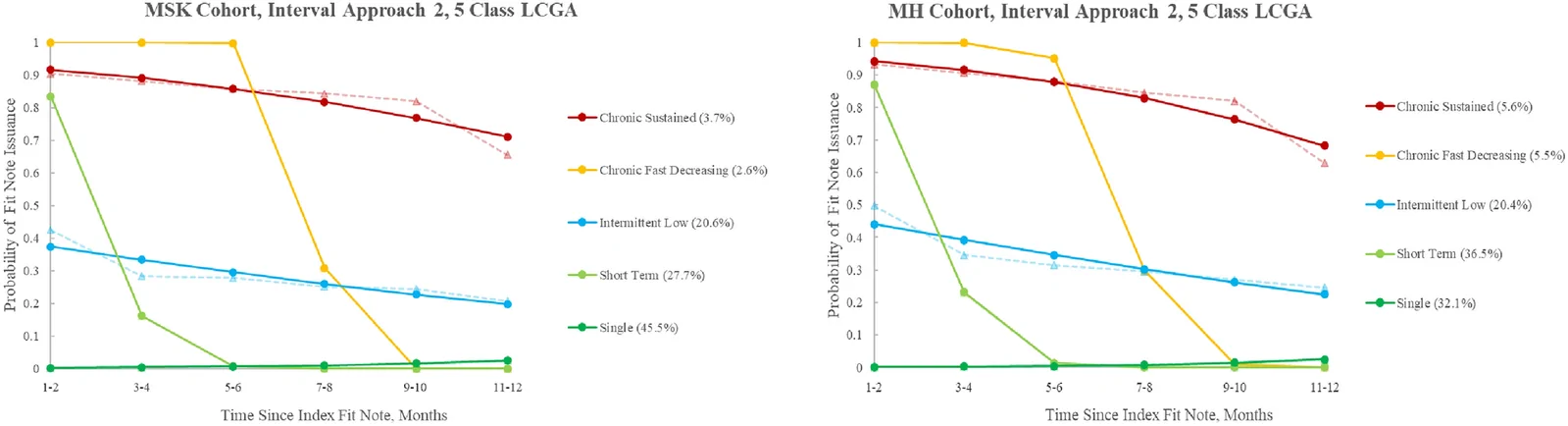
Five-Class LCGA Models, Based on Interval Approach 2 (Two-Monthly Intervals, Year One Data Only), for the Cohort with Index Fit Note Due to a MSK Condition (Left) and MH Condition (Right). *LCGA* latent class growth analysis, *MSK* musculoskeletal, *MH* mental health. For each trajectory, the solid lines represent the probability of fit note issuance in the given time interval (model estimated data), while the dotted lines represent the proportion of individuals issued a fit note (observed data). Trajectory class prevalence is shown, derived from a count based on posterior probability

The optimal LCGA models are displayed graphically in Fig. 1, and corresponding model fit and class meaningfulness statistics presented in Supplementary Materials S3. In particular, these five-class models converged without issues, had a likelihood ratio test *p* value < 0.0001 (based on both the Lo-Mendell-Rubin and the bootstrapped approaches; suggesting the five-class model fitted the data better than a four-class model), and had entropy and average posterior probabilities above our study guideline threshold of 0.7 for all trajectory classes, indicating good class separability.

These optimal models also contained a variety of different and plausible trajectory class shapes that made sense in a work absence context (Fig. 1). The trajectory shapes of these five-class models were similar across the two cohorts and consisted first of two classes characterized by low sickness absence throughout follow-up:

- ‘Single,’ whereby the probability of fit note issuance remained close to 0 for all two-monthly intervals in the first year of follow-up (MSK cohort 45.5% of the population, MH cohort 32.1%). The observed fit note data are consistent with the model predictions (Supplementary Material S4), with around 90% of individuals in the ‘Single’ MSK and MH trajectory classes receiving no fit notes in all six time periods (Tables S.5 and S.6).
- ‘Short Term,’ whereby there was an initial high probability of fit note issuance in the first two months post-index fit note (of around 0.85–0.90), which then decreased sharply to under 0.25 between months three and four of follow-up, and from months five to twelve was close to 0 (MSK 27.7%, MH 36.5%). No individuals in the MSK and MH cohorts had a fit note issued after the second time period (Tables S.5 and S.6).

Then, there were two classes characterized by a high probability of absence:

- ‘Chronic Sustained,’ whereby the probability of fit note issuance remained largely high (between 0.68 and 0.94) throughout all two-monthly intervals in the first year of follow-up (MSK 3.7%, MH 5.6%). Around 90% of individuals in the MSK and MH cohorts had a fit note issued in at least five of the six time periods (Tables S.5 and S.6).
- ‘Chronic Fast Decreasing,’ this trajectory started off similar to the ‘Chronic Sustained’ class, with a high and sustained probability (of close to 1) of fit note issuance in the first six months of follow-up. However, from months seven to eight, there was a rapid decrease to around 0.3 probability of fit note issuance, and then from months nine to twelve, this probability dropped close to 0 (MSK 2.6%, MH 5.5%). In the MSK cohort, 98% of individuals had a fit note issued in each of the first three or four consecutive time periods, and 95% in the MH cohort (Tables S.5 and S.6).

The final derived trajectory was as follows:

– ‘Intermittent Low,’ here the probability of fit note issuance was low, between 0.2 and 0.45 throughout all two-monthly intervals in the first year of follow-up (MSK 20.6%, MH 20.4%). Generally, individuals in this trajectory had either one or two recorded fit notes during the one-year follow-up period (Tables S.7 and S.8).

The variability in observed patterns of fit note issuance over the 12 months follow-up within each of the derived classes was low, except for the ‘Intermittent Low’ trajectory class which was composed of over 80 different types of fit note issuance patterns for both of the MSK and MH cohorts (Tables S.7 and S.8).

### Characteristics Associated with MSK Condition Absence Trajectories

For the MSK cohort, statistically significant adjusted associations common to both of the two most severe absence trajectories (‘Chronic Sustained’ and ‘Chronic Fast Decreasing’) were as follows (Table 3):

- Older age
- Living in the North of England or the Midlands
- Living in the most deprived areas of England
- Having more (≥ 2) prior MSK consultations
- Having baseline knee pain, osteoarthritis, or hip pain, compared to back pain
- Being prescribed opioids, gabapentinoids, or antidepressants in the two years prior to index fit note
- Being current smokers

**Table 3:** Characteristics associated with optimal trajectories of work absence due to a MSK condition using the ‘Single’ trajectory class as the reference (adjusted model)

	Trajectory class			
	Chronic sustained^a^	Chronic fast decreasing^a^	Intermittent low^a^	Short term^a^
	*n* = 1261	*n* = 1333	*n* = 7272	*n* = 11,154
Sex				
Male	1.00 (Ref)	1.00 (Ref)	1.00 (Ref)	1.00 (Ref)
Female	0.93 (0.81, 1.07)	**1.17 (1.01, 1.37)**	**1.15 (1.06, 1.24)**	**1.10 (1.03, 1.17)**
Age				
16–25 years	1.00 (Ref)	1.00 (Ref)	1.00 (Ref)	1.00 (Ref)
26–35 years	**1.38 (1.02, 1.87)**	1.04 (0.79, 1.37)	1.00 (0.88, 1.14)	**1.24 (1.13, 1.36)**
36–45 years	**1.67 (1.25, 2.23)**	1.15 (0.88, 1.50)	**0.84 (0.74, 0.95)**	**1.15 (1.05, 1.26)**
46–55 years	**2.10 (1.59, 2.77)**	**1.42 (1.10, 1.85)**	**0.83 (0.73, 0.93)**	**1.37 (1.24, 1.50)**
56–66 years	**3.26 (2.47, 4.30)**	**2.38 (1.83, 3.10)**	1.04 (0.90, 1.20)	**1.54 (1.38, 1.71)**
Region^b^				
South of England	1.00 (Ref)	1.00 (Ref)	1.00 (Ref)	1.00 (Ref)
North of England	**1.36 (1.16, 1.60)**	**1.70 (1.45, 2.00)**	1.02 (0.92, 1.13)	**1.36 (1.27, 1.46)**
Middle of England	**1.45 (1.24, 1.70)**	**1.24 (1.02, 1.51)**	**1.13 (1.03, 1.25)**	**1.20 (1.11, 1.29)**
IMD^c^				
1 (least deprived)	1.00 (Ref)	1.00 (Ref)	1.00 (Ref)	1.00 (Ref)
2	1.02 (0.78, 1.34)	1.17 (0.90, 1.54)	1.15 (0.99, 1.34)	1.02 (0.92, 1.12)
3	1.22 (0.94, 1.60)	1.19 (0.91, 1.55)	**1.32 (1.15, 1.52)**	1.01 (0.92, 1.12)
4	**1.68 (1.30, 2.16)**	1.27 (0.97, 1.66)	**1.61 (1.41, 1.84)**	0.99 (0.90, 1.09)
5 (most deprived)	**2.50 (1.97, 3.17)**	**1.64 (1.28, 2.09)**	**1.78 (1.55, 2.04)**	0.95 (0.87, 1.05)
Missing	1.22 (0.79, 1.90)	**0.38 (0.15, 0.97)**	0.75 (0.53, 1.07)	**1.30 (1.12, 1.52)**
MSK consultations—prior 2 years^d^				
0	1.00 (Ref)	1.00 (Ref)	1.00 (Ref)	1.00 (Ref)
1	**1.27 (1.07, 1.51)**	1.06 (0.87, 1.29)	1.06 (0.96, 1.17)	**1.09 (1.01, 1.17)**
2	**1.63 (1.30, 2.03)**	**1.70 (1.35, 2.14)**	**1.29 (1.13, 1.47)**	**1.21 (1.09, 1.35)**
≥ 3	**1.81 (1.44, 2.27)**	**1.48 (1.16, 1.90)**	**1.47 (1.29, 1.67)**	**1.30 (1.17, 1.44)**
Baseline MSK condition				
Back pain	1.00 (Ref)	1.00 (Ref)	1.00 (Ref)	1.00 (Ref)
Knee pain	**1.34 (1.11, 1.62)**	**1.47 (1.21, 1.79)**	**1.34 (1.21, 1.49)**	0.95 (0.87, 1.03)
Hand/wrist pain	1.01 (0.72, 1.41)	1.21 (0.87, 1.69)	1.02 (0.86, 1.22)	**0.81 (0.71, 0.93)**
Inflammatory MSK	1.09 (0.80, 1.49)	0.95 (0.66, 1.38)	1.06 (0.86, 1.30)	**0.78 (0.66, 0.92)**
Osteoarthritis	**2.60 (2.00, 3.39)**	**1.84 (1.33, 2.53)**	**2.10 (1.70, 2.59)**	0.87 (0.72, 1.05)
Hip pain	**2.62 (2.00, 3.43)**	**1.68 (1.18, 2.39)**	**1.43 (1.15, 1.78)**	1.13 (0.95, 1.34)
Opioids				
No	1.00 (Ref)	1.00 (Ref)	1.00 (Ref)	1.00 (Ref)
Yes	**1.40 (1.20, 1.65)**	**1.58 (1.32, 1.90)**	**1.18 (1.08, 1.30)**	**1.14 (1.06, 1.23)**
NSAIDs				
No	1.00 (Ref)	1.00 (Ref)	1.00 (Ref)	1.00 (Ref)
Yes	1.16 (1.00, 1.35)	1.08 (0.91, 1.28)	**1.15 (1.05, 1.26)**	1.06 (0.99, 1.14)
Gabapentinoids				
No	1.00 (Ref)	1.00 (Ref)	1.00 (Ref)	1.00 (Ref)
Yes	**1.60 (1.22, 2.11)**	**2.08 (1.58, 2.75)**	**1.51 (1.24, 1.83)**	**1.25 (1.05, 1.49)**
Antidepressants				
No	1.00 (Ref)	1.00 (Ref)	1.00 (Ref)	1.00 (Ref)
Yes	**1.79 (1.52, 2.10)**	**1.48 (1.23, 1.77)**	**1.50 (1.35, 1.67)**	1.07 (0.98, 1.17)
Polypharmacy^e^				
0	1.00 (Ref)	1.00 (Ref)	1.00 (Ref)	1.00 (Ref)
1–4	0.97 (0.80, 1.17)	0.82 (0.67, 1.01)	1.07 (0.96, 1.20)	0.99 (0.92, 1.07)
5–9	1.26 (1.00, 1.59)	1.14 (0.89, 1.45)	**1.36 (1.19, 1.55)**	1.01 (0.91, 1.12)
≥ 10	**1.48 (1.13, 1.95)**	1.37 (1.00, 1.88)	**1.84 (1.54, 2.20)**	1.00 (0.86, 1.15)
Smoking status				
Never	1.00 (Ref)	1.00 (Ref)	1.00 (Ref)	1.00 (Ref)
Current	**1.80 (1.53, 2.10)**	**1.41 (1.19, 1.69)**	**1.36 (1.25, 1.49)**	1.07 (1.00, 1.15)
Ex smoker	**1.24 (1.01, 1.52)**	1.17 (0.94, 1.46)	0.99 (0.87, 1.11)	1.08 (0.99, 1.17)
Not recorded	1.22 (0.95, 1.57)	1.03 (0.79, 1.34)	0.95 (0.83, 1.08)	1.00 (0.91, 1.10)
BMI^f^				
Underweight/normal	1.00 (Ref)	1.00 (Ref)	1.00 (Ref)	1.00 (Ref)
Overweight	0.95 (0.78, 1.17)	0.94 (0.76, 1.18)	**1.16 (1.04, 1.30)**	**1.12 (1.02, 1.22)**
Obese	0.99 (0.82, 1.21)	0.97 (0.78, 1.20)	**1.19 (1.07, 1.33)**	1.05 (0.96, 1.14)
Not recorded	1.20 (0.98, 1.48)	1.11 (0.90, 1.37)	1.07 (0.96, 1.20)	1.06 (0.98, 1.15)
Modified CCI score^g^				
0	1.00 (Ref)	1.00 (Ref)	1.00 (Ref)	1.00 (Ref)
1	**1.34 (1.12, 1.61)**	0.90 (0.72, 1.13)	1.01 (0.90, 1.14)	0.96 (0.88, 1.05)
≥ 2	1.28 (0.95, 1.72)	0.89 (0.62, 1.29)	1.10 (0.91, 1.34)	0.95 (0.80, 1.13)

Values are presented as adjusted odds ratios with 95% confidence intervals (adjustments were made for all the other variables shown in the Table)

*IMD* index of multiple deprivation, *MSK* musculoskeletal, *NSAIDs* non-steroidal anti-inflammatory drugs, *BMI* body mass index, *CCI* Charlson comorbidity index

Statistically significant estimates (where 95% CI doesn’t include the value 1) are shown in bold

^a^All odds ratios are calculated with respect to the reference trajectory: Single

^b^This is defined as the region of the General Practitioner Practice (primary care clinic) of the patient. North of England is defined as follows: Northeast, Northwest, Yorkshire and the Humber; Middle of England: East Midlands, West Midlands, East of England; and South of England: Southeast, Southwest, London

^c^Quintiles are used for IMD (1–5), where a higher score represents more deprived areas. An IMD of 5 represents the most deprived areas of England, and an IMD of 1 the least deprived areas

^d^Excluding the index MSK consultation

^e^Excluding Opioids, NSAIDs, Gabapentinoids, Antidepressants

^f^Underweight/Normal: 10 <  = BMI < 25; Overweight: 25 <  = BMI < 30; Obese: 30 <  = BMI < 80; Not Recorded: no BMI data available or BMI < 10 or BMI >  = 80

^g^Excluding Rheumatic Disease

### Characteristics Associated with MH Condition Absence Trajectories

In the MH cohort, individuals in the two most absence severe trajectories (‘Chronic Sustained’ and ‘Chronic Fast Decreasing’) were more likely to (Table 4):

- Be male
- Be older
- Live in the North of England or the Midlands
- Live in any of the three most deprived areas of England (IMD from 3 to 5)
- Have baseline anxiety, depression, or anxiety and depression combined, compared to stress
- Be prescribed an opioid in the two years prior to index fit note issue
- Have excessive polypharmacy
- Be current smokers
- Be obese or with a ‘not recorded’ BMI

**Table 4:** Characteristics associated with optimal trajectories of work absence due to a MH condition using the ‘Single’ trajectory class as the reference

	Trajectory class			
	Chronic sustained^a^	Chronic fast decreasing^a^	Intermittent low^a^	Short term^a^
	*n* = 2881	*n* = 3848	*n* = 10,835	*n* = 21,534
Sex				
Male	1.00 (Ref)	1.00 (Ref)	1.00 (Ref)	1.00 (Ref)
Female	**0.79 (0.71, 0.87)**	**0.88 (0.80, 0.96)**	1.00 (0.93, 1.07)	0.98 (0.93, 1.03)
Age				
16–25 years	1.00 (Ref)	1.00 (Ref)	1.00 (Ref)	1.00 (Ref)
26–35 years	**0.83 (0.73, 0.95)**	1.05 (0.92, 1.19)	**0.85 (0.78, 0.93)**	**1.38 (1.30, 1.47)**
36–45 years	**1.18 (1.04, 1.35)**	**1.52 (1.33, 1.74)**	**0.79 (0.72, 0.88)**	**1.63 (1.52, 1.74)**
46–55 years	**1.66 (1.43, 1.92)**	**2.17 (1.90, 2.49)**	0.91 (0.82, 1.01)	**1.80 (1.66, 1.94)**
56–66 years	**2.30 (1.92, 2.77)**	**2.72 (2.29, 3.23)**	1.08 (0.94, 1.24)	**1.87 (1.69, 2.08)**
Region^b^				
South of England	1.00 (Ref)	1.00 (Ref)	1.00 (Ref)	1.00 (Ref)
North of England	**1.31 (1.15, 1.49)**	**1.65 (1.48, 1.83)**	0.99 (0.91, 1.08)	**1.34 (1.26, 1.42)**
Middle of England	**1.51 (1.33, 1.72)**	**1.34 (1.20, 1.50)**	0.95 (0.88, 1.03)	**1.20 (1.13, 1.27)**
IMD^c^				
1 (least deprived)	1.00 (Ref)	1.00 (Ref)	1.00 (Ref)	1.00 (Ref)
2	1.21 (1.00, 1.47)	**1.19 (1.01, 1.41)**	1.12 (1.00, 1.26)	0.96 (0.89, 1.04)
3	**1.49 (1.24, 1.79)**	**1.36 (1.16, 1.60)**	**1.28 (1.14, 1.43)**	0.95 (0.88, 1.02)
4	**1.86 (1.55, 2.23)**	**1.49 (1.27, 1.76)**	**1.52 (1.36, 1.71)**	0.95 (0.88, 1.03)
5 (most deprived)	**2.67 (2.24, 3.18)**	**2.00 (1.71, 2.33)**	**1.83 (1.64, 2.04)**	0.93 (0.86, 1.01)
Missing	**0.68 (0.48, 0.95)**	0.93 (0.72, 1.19)	**0.54 (0.41, 0.71)**	1.11 (0.99, 1.25)
MH consultations—prior 2 years^d^				
0	1.00 (Ref)	1.00 (Ref)	1.00 (Ref)	1.00 (Ref)
1	1.03 (0.90, 1.17)	1.07 (0.94, 1.22)	1.08 (0.98, 1.18)	0.97 (0.91, 1.04)
2	1.17 (0.98, 1.41)	1.14 (0.95, 1.36)	1.12 (0.99, 1.26)	0.99 (0.90, 1.09)
≥ 3	**1.20 (1.02, 1.42)**	1.06 (0.91, 1.25)	**1.28 (1.15, 1.43)**	0.94 (0.86, 1.03)
Baseline MH condition				
Stress	1.00 (Ref)	1.00 (Ref)	1.00 (Ref)	1.00 (Ref)
Anxiety and depression	**3.72 (3.17, 4.36)**	**3.31 (2.89, 3.80)**	**1.69 (1.53, 1.86)**	**1.63 (1.52, 1.75)**
Depression	**3.32 (2.85, 3.87)**	**2.77 (2.42, 3.18)**	**1.55 (1.40, 1.70)**	**1.50 (1.40, 1.60)**
Anxiety	**2.04 (1.74, 2.38)**	**1.56 (1.35, 1.80)**	**1.19 (1.09, 1.31)**	**1.13 (1.06, 1.21)**
Opioids				
No	1.00 (Ref)	1.00 (Ref)	1.00 (Ref)	1.00 (Ref)
Yes	**1.37 (1.20, 1.56)**	**1.16 (1.01, 1.33)**	**1.33 (1.21, 1.46)**	0.97 (0.89, 1.04)
NSAIDs				
No	1.00 (Ref)	1.00 (Ref)	1.00 (Ref)	1.00 (Ref)
Yes	1.06 (0.92, 1.22)	0.97 (0.85, 1.11)	1.04 (0.95, 1.14)	1.02 (0.95, 1.10)
Gabapentinoids				
No	1.00 (Ref)	1.00 (Ref)	1.00 (Ref)	1.00 (Ref)
Yes	0.98 (0.73, 1.32)	0.87 (0.62, 1.22)	1.14 (0.91, 1.43)	0.82 (0.67, 1.01)
Antidepressants				
No	1.00 (Ref)	1.00 (Ref)	1.00 (Ref)	1.00 (Ref)
Yes	1.09 (0.97, 1.23)	0.95 (0.85, 1.07)	**1.25 (1.14, 1.36)**	**0.85 (0.80, 0.90)**
Polypharmacy^e^				
0	1.00 (Ref)	1.00 (Ref)	1.00 (Ref)	1.00 (Ref)
1–4	0.93 (0.82, 1.06)	0.93 (0.83, 1.04)	1.05 (0.95, 1.15)	1.00 (0.94, 1.07)
5–9	1.03 (0.88, 1.21)	1.01 (0.87, 1.17)	**1.22 (1.09, 1.36)**	0.99 (0.92, 1.07)
≥ 10	**1.27 (1.02, 1.59)**	**1.29 (1.05, 1.59)**	**1.59 (1.36, 1.86)**	0.96 (0.85, 1.09)
Smoking status				
Never	1.00 (Ref)	1.00 (Ref)	1.00 (Ref)	1.00 (Ref)
Current	**1.75 (1.56, 1.95)**	**1.53 (1.38, 1.70)**	**1.44 (1.33, 1.55)**	1.05 (0.99, 1.11)
Ex smoker	0.99 (0.84, 1.17)	0.99 (0.85, 1.14)	1.06 (0.96, 1.18)	1.04 (0.96, 1.11)
Not recorded	1.17 (1.00, 1.38)	1.11 (0.96, 1.28)	**1.20 (1.08, 1.33)**	**1.09 (1.01, 1.17)**
BMI^f^				
Underweight/Normal	1.00 (Ref)	1.00 (Ref)	1.00 (Ref)	1.00 (Ref)
Overweight	1.00 (0.87, 1.16)	0.97 (0.85, 1.11)	0.97 (0.88, 1.07)	1.04 (0.97, 1.11)
Obese	**1.28 (1.11, 1.48)**	**1.21 (1.05, 1.39)**	**1.19 (1.08, 1.31)**	**1.12 (1.04, 1.21)**
Not recorded	**1.22 (1.07, 1.39)**	**1.18 (1.04, 1.33)**	1.06 (0.97, 1.15)	1.00 (0.94, 1.06)
CCI score				
0	1.00 (Ref)	1.00 (Ref)	1.00 (Ref)	1.00 (Ref)
1	1.14 (0.99, 1.31)	0.96 (0.83, 1.10)	**1.21 (1.09, 1.33)**	0.97 (0.90, 1.05)
≥ 2	1.10 (0.84, 1.43)	0.99 (0.76, 1.29)	**1.34 (1.10, 1.63)**	0.98 (0.84, 1.15)

Values are presented as adjusted odds ratios with 95% confidence intervals (adjustments were made for all the other variables in the Table)

*IMD* index of multiple deprivation, *MH* mental health, *NSAIDs* non-steroidal anti-inflammatory drugs, *BMI* body mass index, *CCI* Charlson comorbidity index

Statistically significant estimates (where 95% CI does not include the value 1) are shown in bold

^a^All odds ratios are calculated with respect to the reference trajectory: Single

^b^This is defined as the region of the General Practitioner Practice (primary care clinic) of the patient. North of England is defined as follows: Northeast, Northwest, Yorkshire and the Humber; Middle of England: East Midlands, West Midlands, East of England; and South of England: Southeast, Southwest, London

^c^Quintiles are used for IMD (1–5), where a higher score represents more deprived areas. An IMD of 5 represents the most deprived areas of England, and an IMD of 1 the least deprived areas

^d^Excluding the index MH consultation

^e^Excluding Opioids, NSAIDs, Gabapentinoids, Antidepressants

^f^Underweight/Normal: 10 <  = BMI < 25; Overweight: 25 <  = BMI < 30; Obese: 30 <  = BMI < 80; Not Recorded: no BMI data available or BMI < 10 or BMI >  = 80

## Discussion

### Summary of Main Findings and Comparison with Other Research

In this large study of trajectories of work absence in England, five common trajectories associated with MSK and MH conditions were determined over a one-year follow-up. The two most prevalent trajectories consisted of low absence (issuance of either a ‘Single’ index fit note or a ‘Short Term’ absence lasting two to four months); these two trajectories comprised 73.2% and 68.6% of economically active individuals in England who experienced a first absence due to a MSK or MH condition, respectively.

In contrast, our two least prevalent trajectories were characterized by longer-term absence lasting six months or more (‘Chronic Sustained’ or ‘Chronic Fast Decreasing’ absence); these two patterns of sickness absence were experienced by 6.3% and 11.1% of our study MSK and MH cohort populations, respectively. This is concerning, as long-term sickness absence has risen to record numbers and is the main reason for economic inactivity in the UK (currently accounting for 30.2% of the economically inactive population) [1, 21]. This puts the UK economy at a disadvantage compared to those of other Western countries whose economies have since shown recovery toward pre-pandemic levels [22].

The fifth trajectory subgroup, ‘Intermittent Low’ (prevalence 20% in both MSK and MH cohorts), had a less clearly identifiable pattern other than being episodic fit notes, suggesting a subgroup of individuals who were in-and-out of absence during the one-year follow-up.

The findings that trajectories of longer absence occur with lower prevalence, and trajectories of less severe absence occur with higher prevalence, have also been demonstrated in other studies [7, 8, 23–25]. Furthermore, similar shapes of the five trajectories we identified have also been found in other studies. For example, similar trajectories involving a sustained high level of work absence throughout follow-up, analogous to our most severe ‘Chronic Sustained’ class (3.7% and 5.6% prevalence, for the MSK and MH condition fit note cohorts, respectively), have been identified by Farrants et al. (2019) [7] in a Swedish population (a ‘Late Decrease’ class, 8.0%), Rysstad et al. (2023) [8] in a Norwegian population (a ‘Persistent High’ class, 18.2% prevalence), and McLeod et al. (2018) [23] in a Canadian population (a ‘Constant Sickness Absence’ class, 3.0% prevalence). Our study was conducted in a UK population where there is a 7-day self-certification period before a medically certified sickness absence is required to be able to claim sick pay. A similar 7-day self-certification absence system is in place in countries such as Sweden [26, 27] and Norway (which has a minimum self-certification period of 3 days, although many employers allow up to 8 days [28]). While absence management systems can otherwise vary internationally [29], a systematic review by Kausto et al. (2019) [30] found inconclusive evidence linking differing sickness absence management systems and the duration or frequency of absence spells; further research is needed to better understand this.

Finally, we found that a set of common characteristics were associated with both of our two longer-term absence trajectories (‘Chronic Sustained’ and ‘Chronic Fast Decreasing’); individuals who were older, living in the North or Midlands or more deprived areas of England, prescribed opioids in the two years preceding their index fit note, and current smokers. Older individuals have also been shown to be associated with longer-term absence trajectories in other studies by Farrants et al. (2019) [7], Rysstad et al. (2023) [8], Farrants et al. (2018) [24], and Spronken et al. (2020) [25].

Our trajectory-covariate association analysis also highlighted the presence of health inequalities. For example, those from the most deprived neighborhoods were more likely to follow one of the two longer-term absence trajectories. Health inequality by deprivation status has been also demonstrated by Marmot et al. (2010) [31] who showed in their Strategic Review of Health Inequalities in England post-2010 report that those in the most deprived neighborhoods of England, compared to the least deprived, lived shorter lives and with more disability. More recently, Parker et al. (2020) [32] showed that these people from worse off areas of England also had a lower healthy working life expectancy than those from better off areas.

The key findings from this study, identifying profiles of individuals most at risk of longer-term sickness absence due to a MSK or MH condition, provide useful evidence to target upcoming Government initiatives to those who need support the most (such as those from more economically deprived areas of England). These Government incentives include WorkWell [22], a low-intensity, holistic early intervention of work and health support designed to help people RTW, funded in 15 different areas across England. Additionally, the fit note reform [33] is a Government initiative to evaluate the state of the current fit note process, with the aim to improve access to timely health and work support.

Alongside Government initiatives, policy to incentivise employers for engaging in training concerning RTW management of their employees may also be beneficial, to help employers become more accommodating and supportive of their employees’ needs, as well as broader policies to reduce national health inequalities [31].

### Study Strengths and Limitations

One of the key strengths of this study was the use of a large, nationally representative primary care database [9]. A further key strength was that high-quality trajectory reporting was conducted using the GRoLTS checklist [17], and a variety of statistical measures were used to guide the choice of the optimal models, to ensure robustness of the final selection. Face validity of the findings from this study was affirmed through discussions with a wide range of stakeholders including General Practitioners, our patient and public involvement and engagement group, and based on feedback from meetings which included members of the Office for Health Improvement and Disparities, the Department for Work and Pensions, and Versus Arthritis.

However, a limitation was that it was not possible to perform a trajectory derivation analysis based on duration of fit note as this was largely missing in the formatted primary care data available for analysis. This may lead to some misrepresentation of absence patterns. For example, individuals may have had multiple (‘short’) absence spells in a given two-month time interval; here, our approach would ignore such complexity and simply count this as one absence. However, this is likely to be uncommon given 50% of all fit notes issued in England due to a MSK or MH condition are not ‘short’ and have a duration of at least one month [12], hence why we chose to examine absence intervals of at least two months duration. Conversely, longer absence spells (lasting more than two months) may have been underrepresented with our approach. But, as we show in Supplementary Materials S2, by reducing time intervals in trajectory derivation models from six to three to two months, the performance of the models improved progressively, and the two-month choice of intervals was therefore deemed optimal. Furthermore, plausibility and face validity of our derived trajectories were affirmed from key stakeholders, and similar shaped trajectories to ours have been observed in other international studies. Had a continuous fit note definition been possible to examine in this study, this may have led to models with an increased complexity which may have more convergence issues and be more difficult to interpret, and therefore, such models may be less practical than the ones we developed.

We could have considered other trajectory derivation methods also, such as growth mixture modeling (GMM), which assumes that there are variations between people within trajectories but is more complex than LCGA. Analyses not presented here investigated using a five-class GMM model for the incident MSK condition fit note cohort (to compare against our optimal five-class LCGA model), but this GMM model exhibited excessive computational burden and convergence issues, including possibly unreliable solutions and it was not clear whether a local maxima solution had been achieved in the GMM model rather than a global solution. Nonetheless, despite these issues, the five-class GMM model derived similar trajectory shapes and prevalences to the optimal five-class LCGA model (Figure S.1 in Supplementary Materials S5); therefore, further exploration of GMM was not deemed necessary.

Our models were based on issued fit notes and not workplace data. A limitation of using primary care electronic health records is that workplace and RTW data are not available. We may have therefore missed some missed some potentially important covariates when testing for association with our derived absence trajectories. For example, self-efficacy to RTW is important as individuals’ recovery expectations have been identified in other studies as a strong predictor of RTW [34–37]. Negative RTW expectancy was one of the most important predictors discovered in the study by Rysstad et al. [8], that was associated with all three of their trajectories that involved the most sickness absence days. Furthermore, it would be useful to assess the impact of work-related characteristics on association with longer-term absence trajectories, including full time compared to part-time work, type of work (unskilled, manual, skilled etc.), and level of job control. Having access to such data might further identify specific subgroups that are at greater risk of intermittent or long-term absence and that could benefit from early and more targeted health and work support after onset of an initial sickness absence.

Finally, as reason for fit note was not available in the formatted primary care electronic health records used in this study, we assumed that the index fit note was due to a MSK or MH condition if there was a MSK or MH consultation, respectively, up to two weeks prior to or on the first ever fit note date. It is not expected that the true number of MSK and MH condition fit notes was substantially underestimated, as, of the index fit notes analyzed in this study, over 85% had MSK and MH consultations that occurred on the day of the index fit note. Nonetheless, it would be useful to validate our results using reason for absence data taken directly from a fit note, if it becomes more possible to access this in the future. However, reason for absence taken directly from a fit note can also be limiting; Dorrington et al. (2021) [38] argued that many fit notes do not provide sufficient information for a sickness absence reason to be properly coded into an EHR database. Also, the information on the fit note might not always depict the true reason for sickness absence, for example, if a patient is sensitive to information about a MH problem being shared with their employer, the healthcare professional might write another reason on the fit note.

### Recommendations for Future Research

Further external validation of these trajectories would be valuable, including internationally. Qualitative work may also help to better understand what kind of support might benefit those in or at risk of the more severe trajectories of long-term sickness absence. Such a study could aim to understand what barriers these people face to RTW, and what support could help them reach a sustained RTW more quickly.

## Conclusion

Using a representative sample of economically active English workers experiencing a first ever sickness absence due to a MSK or MH condition, long-term sickness absence patterns lasting six months or more occurred in 6.3% and 11.1% of the MSK and MH cohorts, respectively. Health inequalities were demonstrated; individuals associated with these longer-term absence patterns were older, living in the North or Midlands or most deprived areas of England, prescribed opioids, and current smokers. These findings have implications for prevention and management strategies for individuals experiencing an incident work absence due to either a MSK or MH condition.

## Supplementary Information

Below is the link to the electronic supplementary material.

Supplementary file1 (DOCX 258 KB)

## Data Availability

Data may be obtained from a third party and are not publicly available. The data were obtained from the Clinical Practice Research Datalink. Clinical Practice Research Datalink data governance does not allow us to distribute patient data to other parties. Researchers may apply for data access at http://www.CPRD.com/.
