# Supplementary material for: Trajectories of Work Absence in England due to a Musculoskeletal or Mental Health Condition: An Electronic Health Record Cohort Study
Source: J Occup Rehabil. 2025 Nov 28;36(4):1155–67. doi: 10.1007/s10926-025-10342-y (PMC13759608; doi:10.1007/s10926-025-10342-y)
Supplement: Supplementary file 1 [file 10926_2025_10342_MOESM1_ESM.docx]

# Supplementary Material

# S1: Descriptive Summary Table of Included MSK and MH Condition Cohorts

**Table S.1.** Descriptive Statistics of Cohorts with an Index Fit Note Due to Either a MSK or MH Condition

|  | **Cohort** | |
| --- | --- | --- |
|  | **MSK Condition Index Fit Note** | **MH Condition Index Fit Note** |
|  | **n=43,130** | **n=62,355** |
| **Female** | 19637 (45.53%) | 36937 (59.24%) |
| **Age** |  |  |
| 16-25 years | 6897 (15.99%) | 17271 (27.70%) |
| 26-35 years | 9073 (21.04%) | 16700 (26.78%) |
| 36-45 years | 9477 (21.97%) | 12952 (20.77%) |
| 46-55 years | 10503 (24.35%) | 10562 (16.94%) |
| 56-66 years | 7180 (16.65%) | 4870 (7.81%) |
| **Region^a^** |  |  |
| North of England | 10475 (24.29%) | 19310 (30.97%) |
| Middle of England | 10185 (23.61%) | 14487 (23.23%) |
| South of England | 22470 (52.10%) | 28558 (45.80%) |
| **IMD^b^** |  |  |
| 1 (least deprived) | 6008 (13.93%) | 9903 (15.88%) |
| 2 | 7003 (16.24%) | 10623 (17.04%) |
| 3 | 7780 (18.04%) | 10834 (17.37%) |
| 4 | 9763 (22.64%) | 12514 (20.07%) |
| 5 (most deprived) | 11018 (25.55%) | 15627 (25.06%) |
| Missing | 1558 (3.61%) | 2854 (4.58%) |
| **MSK or MH Consultations - Prior 2 Years^c^** |  |  |
| 0 | 21642 (50.18%) | 36827 (59.06%) |
| 1 | 11135 (25.82%) | 10925 (17.52%) |
| 2 | 4705 (10.91%) | 5225 (8.38%) |
| ≥3 | 5648 (13.10%) | 9378 (15.04%) |
| **Baseline MSK Condition** |  |  |
| Back pain | 30232 (70.10%) | N/A |
| Knee pain | 6424 (14.89%) | N/A |
| Hand/wrist pain | 2127 (4.93%) | N/A |
| Inflammatory MSK | 1532 (3.55%) | N/A |
| Osteoarthritis | 1509 (3.50%) | N/A |
| Hip pain | 1306 (3.03%) | N/A |
| **Baseline MH Condition** |  |  |
| Stress | N/A | 16339 (26.20%) |
| Anxiety and Depression | N/A | 14769 (23.69%) |
| Depression | N/A | 15695 (25.17%) |
| Anxiety | N/A | 15552 (24.94%) |
| **Opioids** | 13117 (30.41%) | 8139 (13.05%) |
| **NSAIDs** | 13256 (30.73%) | 8023 (12.87%) |
| **Gabapentinoids** | 1823 (4.23%) | 1122 (1.80%) |
| **Antidepressants** | 6883 (15.96%) | 24934 (39.99%) |
| **Polypharmacy^d^** |  |  |
| 0 | 9404 (21.80%) | 12927 (20.73%) |
| 1-4 | 21008 (48.71%) | 31262 (50.14%) |
| 5-9 | 8880 (20.59%) | 13375 (21.45%) |
| ≥10 | 3838 (8.90%) | 4791 (7.68%) |
| **Smoking Status** |  |  |
| Never | 16899 (39.18%) | 23727 (38.05%) |
| Current | 13961 (32.37%) | 21566 (34.59%) |
| Ex Smoker | 6095 (14.13%) | 8171 (13.10%) |
| Not Recorded | 6175 (14.32%) | 8891 (14.26%) |
| **BMI^e^** |  |  |
| Underweight/Normal | 9010 (20.89%) | 17637 (28.28%) |
| Overweight | 9597 (22.25%) | 11454 (18.37%) |
| Obese | 9093 (21.08%) | 10218 (16.39%) |
| Not Recorded | 15430 (35.78%) | 23046 (36.96%) |
| **CCI Score^f^** |  |  |
| 0 | 35839 (83.10%) | 52355 (83.96%) |
| 1 | 5763 (13.36%) | 8323 (13.35%) |
| ≥2 | 1528 (3.54%) | 1677 (2.69%) |

Values are presented as n (%)

Abbreviations: N/A = Not Applicable; IMD = Index of Multiple Deprivation; MSK = Musculoskeletal; MH = Mental Health; NSAIDs = Non-Steroidal Anti-Inflammatory Drugs; BMI = Body Mass Index; CCI = Charlson Comorbidity Index

^a^ This is defined as the region of the General Practitioner Practice (primary care clinic) of the patient. North of England is defined as: North East, North West, Yorkshire and the Humber; Middle of England: East Midlands, West Midlands, East of England; and South of England: South East, South West, London

^b^ Quintiles are used for IMD (1-5), where a higher score represents more deprived areas. An IMD of 5 represents the most deprived areas of England, and an IMD of 1 the least deprived areas

^c^ For the MSK cohort, the number of prior MSK consultations are presented, and for the MH cohort the number of prior MH consultations. The index MSK and MH consultations are excluded

^d^ Excluding Opioids, NSAIDs, Gabapentinoids, Antidepressants

^e^ Underweight/Normal: 10<=BMI<25; Overweight: 25<=BMI<30; Obese: 30<=BMI<80; Not Recorded: no BMI data available or BMI<10 or BMI>=80

^f^ For MSK cohort only, a modified CCI is presented that excludes Rheumatic Disease

# S2: Choosing an Optimal Trajectory LCGA Model

As will be explained in this Section, a five-class LCGA model using approach 2 (one year follow-up with two-monthly intervals) was chosen as the optimal LCGA model, for both the MSK and MH condition fit note cohorts.

This decision was reached taking into consideration the trajectory derivation results across all five interval approaches and our model assessment guidelines. Furthermore, this decision was made through consensus between AL and his co-authors after reflecting on conversations with clinicians, patient and public involvement and engagement group members, and based on feedback from meetings which included members of the Office for Health Improvement and Disparities, the Department for Work and Pensions, and Versus Arthritis.

In the first step of the decision-making process, the general performance of the LCGA models under each of the five interval approaches was first assessed (summarised in Tables S.2 and S.3, for the MSK and MH condition fit note analyses, respectively).

For both the MSK and MH condition fit note trajectory derivation analysis, approaches 1, 3, and 4 (involving either three- and/or six-monthly recurring intervals) performed worst. These three approaches exhibited the lowest entropy values, either close to or below our guideline threshold of 0.7. In some cases, the entropy was considerably lower than the guideline threshold (for example, the highest entropy value under approach 3 was 0.57 for the index MSK condition fit note cohort), suggesting possible class separability issues. In contrast, the entropy values of the LCGA models fit under approaches 2 and 5 (which featured two-monthly intervals) were higher.

**Table S.2.** Summary of LCGA Trajectory Models Based on Individuals with an Index Fit Note Due to a MSK Condition

| **Approach** | **Follow-up**  **Length** | **Year One Follow-Up Time Interval** | **Years Two-Three Time Interval Length** | **Highest Number of Classes Used in LCGA Models** | **Highest Entropy Value** |
| --- | --- | --- | --- | --- | --- |
| 1 (Short-Term) | One Year | Three-Monthly | N/A | 3 | 0.66 |
| 2 (Short-Term) | One Year | Two-Monthly | N/A | 5 | 0.72 |
| 3 (Long-Term) | Three Years | Six-Monthly | Six-Monthly | 3 | 0.57 |
| 4 (Long-Term) | Three Years | Three-Monthly | Six-Monthly | 2^a^ | 0.70 |
| 5 (Long-Term) | Three Years | Two-Monthly | Six-Monthly | 6 | 0.75 |

Abbreviations: LCGA = Latent Class Growth Analysis; MSK = Musculoskeletal; N/A = Not Applicable
^a^ This is based on piecewise LCGA, as the non-piecewise two-class LCGA did not run successfully under this interval approach

**Table S.3.** Summary of LCGA Trajectory Models Based on Individuals with an Index Fit Note Due to a MH Condition

| **Approach** | **Follow-up Length** | **Year One  Time Interval Length** | **Years Two-Three Time Interval Length** | **Highest Number of Classes Used in LCGA Models** | **Highest Entropy Value** |
| --- | --- | --- | --- | --- | --- |
| 1 | One Year | Three-Monthly | N/A | 3 | 0.73 |
| 2 | One Year | Two-Monthly | N/A | 6 | 0.77 |
| 3 | Three Years | Six-Monthly | Six-Monthly | 3 | 0.68 |
| 4 | Three Years | Three-Monthly | Six-Monthly | 3^a^ | 0.72 |
| 5 | Three Years | Two-Monthly | Six-Monthly | 6 | 0.75 |

Abbreviations: LCGA = Latent Class Growth Analysis; MH = Mental Health; N/A = Not Applicable  ^a^ This is based on piecewise LCGA, as the non-piecewise two-class LCGA did not run successfully under this interval approach

Furthermore, there was a lack of variability in the derived trajectories based on approaches 1, 3 and 4 - only a maximum of three classes were uncovered. Whilst under approaches 2 and 5, not only were the trajectories from approaches 1, 3 and 4 replicated, but additional trajectory classes were also uncovered.

Therefore, due to inferior class separability performance and fewer derived trajectories, LCGA models relating to approaches 1, 3 and 4 were excluded from consideration for the optimal model.

Then, of the two remaining approaches (2 and 5), approach 5 was also ruled out. The rationale for this, first for the MSK condition index fit note cohort, was through comparing the LCGA plots from approach 5 that are based on three-year follow-up, to those of approach 2 based on one year follow-up. Whilst there were some similarities in the classes derived, a notable difference was that the trajectories from approach 5 were more prone to poor graphical fit compared to the observed data, whereas those of approach 2 generally showed good fit. The same trend was observed for the MH condition index fit note cohort (comparing the plots from approach 5 to those of approach 2).

Therefore, approach 2 (two-monthly recurring intervals for year one data only) was considered the optimal interval approach in this study, for both the MSK and MH index fit note cohorts.

Then, first considering the index MSK condition fit note cohort, the optimal model choice was narrowed down to either the four- or five-class LCGA within approach 2. Both of these models exhibited good performance, with:

- The lowest and similar AIC and BIC values
- Statistically significant *p* values from likelihood ratio tests

These model fit index values suggested good model fit, which was further affirmed from the trajectory class plots, albeit the ‘Intermittent Low’ and ‘Chronic Sustained’ classes (present in both the four- and five-class LCGA models) showed some instances of poor visual model fit.

Additionally, meaningfulness of classes in these four- and five-class LCGA models was demonstrated by:

- Entropy and average posterior probability values ≥0.7 for all classes, suggesting good class separability
- Minimum prevalence ≥1% for all classes, thereby reducing the risk of spurious results being discovered
- A variety of different and plausible trajectory class shapes, that made sense in a work absence context

Ultimately, for the MSK condition index fit note cohort, it was decided that the five-class LCGA under interval approach 2 was the most appropriate choice (Figure 1). This decision was made considering clinical relevance and plausibility of the classes. In particular, the five-class LCGA provided a clear distinction between the subgroups who had a ‘Short Term’ sickness absence episode, compared to a ‘Single’ index fit note, unlike the four-class LCGA. A GP (VKW) also confirmed that these two ‘Short Term’ and ‘Single’ subgroups were common in her experience of issuing fit notes.

Additionally, the ‘Chronic Fast Decreasing’ class that was present in the five-class LCGA, and not in the four-class model, seemed reasonable in the context of UK sick pay. Statutory sick pay in the UK, is available for eligible claimants for a period of up to twenty-eight weeks [1]. After this point, additional remuneration may be claimable following another evaluation. For example, an application can be made to claim benefits such as the Employment and Support Allowance or Universal Credit.^1^ Therefore, the sharp decrease in probability of fit note issuance after six months of follow-up observed in this trajectory class may be influenced by the original statutory sick pay period ending.

Finally, for the index MH condition fit note cohort, from the two-monthly recurring interval LCGAs under approach 2, the five-class LCGA was again chosen as the optimal model (Figure 1). In the decision-making process, the four-, five- and six-class LCGAs were all contenders for the optimal model, for the same reasons as given for models based on incidence absence due to a MSK condition.

In particular, the six-class model contained the previously mentioned novel ‘Single with Later Relapse’ class (2.9% prevalence). This ‘Single with Later Relapse’ class was also a subgroup of clinical interest highlighted by VKW. Furthermore, this six-class LCGA differentiated between two types of ‘Chronic Fast Decreasing’ class, with a novel ‘Chronic Long, Fast Decreasing’ class (2.7% prevalence).

However, both of these two classes occurred with low prevalences, and were not observed in any other LCGA models in this study. Hence there was a possibility that the uncovering of these two novel classes could be spurious findings.

Additionally, in consideration of parsimony, as well as consistency with the MSK fit note final model, it was decided to reject the six-class model and choose the five-class LCGA as the optimal model for the MH fit note analysis (Figure 1).

**References**

1.	UK Government. Statutory Sick Pay (SSP). Accessed October 20, 2023. https://www.gov.uk/statutory-sick-pay

# S3: Statistical Performance Table of Optimal MSK and MH Condition Trajectory Models

**Table S.4.** Model Fit and Class Meaningfulness Statistics of Optimal Five-Class LCGA Models for MSK and MH Cohort, Interval Approach 2 (Two Monthly Intervals, Year One Data Only)

|  | **Cohort** | |
| --- | --- | --- |
|  | **MSK Condition Index Fit Note** | **MH Condition Index Fit Note** |
|  | **n=43,130** | **n=62,355** |
| **Log-likelihood** | -87417 | -143694 |
| **AIC** | 174863 | 287415 |
| **BIC** | 174984 | 287542 |
| **Likelihood Ratio Test^a^** | p <0.0001 | p <0.0001 |
| **Average Posterior Probability (Range)** | 0.79-0.90 | 0.81-0.91 |
| **Entropy** | 0.72 | 0.73 |
| **Trajectory Class Prevalence** | 45.5%, 27.7%, 20.6%, 3.7%, 2.6% | 36.5%, 32.1%, 20.4%, 5.6%, 5.5% |

Abbreviations: n = number of individuals; LCGA = Latent Class Growth Analysis; MSK = Musculoskeletal; MH = Mental Health; AIC = Akaike Information Criterion; BIC = Bayesian Information Criterion
^a^ The Likelihood Ratio Test was assessed with both the Lo-Mendell-Rubin and bootstrapped methods

# S4: Assessing the Individual Variability Within Each of the Trajectory Classes for the Optimal Five-Class LCGA Models Under Approach 2

**Table S.5.** Summary of Observed Fit Note Issuance Patterns During Year One Follow-Up for the Trajectory Classes in the Optimal Five-Class LCGA Model for the Incident MSK Condition Fit Note Cohort, Excluding the ‘Intermittent Low’ Trajectory Class

| **Trajectory Class** | **Outcome Pattern  (2 monthly)^a^** | **n (%)^b^** | **Median Intervals With Fit Note Received** |
| --- | --- | --- | --- |
| **Chronic Sustained** | 1,1,1,1,1,1 | 485 (38.46%) | 5 |
|  | 1,1,1,1,1,0 | 290 (23.00%) |  |
|  | 1,1,1,1,0,1 | 88 (6.98%) |  |
|  | 1,1,1,0,1,1 | 80 (6.34%) |  |
|  | 1,1,0,1,1,1 | 75 (5.95%) |  |
|  | 1,1,1,0,1,0 | 72 (5.71%) |  |
|  | 0,1,1,1,1,1 | 54 (4.28%) |  |
|  | 1,0,1,1,1,1 | 50 (3.97%) |  |
|  | 1,1,1,.,.,. | 37 (2.93%) |  |
|  | 1,1,1,1,.,. | 17 (1.35%) |  |
| **Chronic Fast Decreasing** | 1,1,1,0,0,0 | 883 (66.24%) | 3 |
|  | 1,1,1,1,0,0 | 421 (31.58%) |  |
|  | 1,1,1,1,0,. | 11 (0.83%) |  |
|  | 1,1,1,0,0,. | 10 (0.75%) |  |
|  | 1,1,1,0,.,. | 8 (0.60%) |  |
| **Short Term** | 1,0,0,0,0,0 | 8544 (76.60%) | 1 |
|  | 1,1,0,0,0,0 | 1845 (16.54%) |  |
|  | 1,.,.,.,.,. | 193 (1.73%) |  |
|  | 1,0,0,.,.,. | 122 (1.09%) |  |
|  | 1,0,.,.,.,. | 116 (1.04%) |  |
|  | 1,0,0,0,.,. | 111 (1.00%) |  |
|  | 1,0,0,0,0,. | 98 (0.88%) |  |
|  | 1,1,.,.,.,. | 56 (0.50%) |  |
|  | 1,1,0,.,.,. | 30 (0.27%) |  |
|  | 1,1,0,0,.,. | 24 (0.22%) |  |
|  | 1,1,0,0,0,. | 15 (0.13%) |  |
| **Single** | 0,0,0,0,0,0 | 20117 (90.99%) | 0 |
|  | 0,0,0,0,0,1 | 737 (3.33%) |  |
|  | 0,0,0,.,.,. | 261 (1.18%) |  |
|  | 0,0,.,.,.,. | 260 (1.18%) |  |
|  | 0,0,0,0,.,. | 260 (1.18%) |  |
|  | 0,.,.,.,.,. | 256 (1.16%) |  |
|  | 0,0,0,0,0,. | 219 (0.99%) |  |

Abbreviations: LCGA = Latent Class Growth Analysis; MSK = Musculoskeletal.

^a^ A value of 1 is used to denote that at least one fit note was issued in the given time interval, 0 denotes that no fit notes were issued, and a period indicates that the individual had missing data in the time interval.

^b^ Cell counts less than five are not shown in accordance with CPRD reporting guidelines (to reduce risk of patient identification).

The pattern is chronologically ordered in two monthly time intervals during the first year of follow-up since index fit note. For example, a pattern of 1,0,0,0,0,0 indicates that a fit note was issued in the first two months since index fit note, but not in the ensuing 10 months.

Note: trajectory prevalence is based on most likely latent class membership, not posterior probabilities, as individuals are treated as whole persons in the observed data, hence the posterior probabilities cannot be used.

**Table S.6.** Summary of Observed Fit Note Issuance Patterns During Year One Follow-Up for the Trajectory Classes in the Optimal Five-Class LCGA Model for the Incident MH Condition Fit Note Cohort, Excluding the ‘Intermittent Low’ Trajectory Class

| **Trajectory Class** | **Outcome Pattern  (2 monthly)^a^** | **n (%)^b^** | **Median Intervals With Fit Note Received** |
| --- | --- | --- | --- |
| **Chronic Sustained (4.6%)** | 1,1,1,1,1,1 | 1061 (36.83%) | 5 |
|  | 1,1,1,1,1,0 | 734 (25.48%) |  |
|  | 1,1,1,1,0,1 | 208 (7.22%) |  |
|  | 1,1,1,0,1,1 | 203 (7.05%) |  |
|  | 1,1,1,0,1,0 | 192 (6.66%) |  |
|  | 1,1,0,1,1,1 | 152 (5.28%) |  |
|  | 1,0,1,1,1,1 | 122 (4.23%) |  |
|  | 0,1,1,1,1,1 | 111 (3.85%) |  |
|  | 1,1,1,1,.,. | 58 (2.01%) |  |
|  | 1,1,1,1,1,. | 22 (0.76%) |  |
|  | 1,1,1,0,1,. | 9 (0.31%) |  |
| **Chronic Fast Decreasing (6.2%)** | 1,1,1,0,0,0 | 2479 (64.42%) | 3 |
|  | 1,1,1,1,0,0 | 1166 (30.3%) |  |
|  | 1,1,1,.,.,. | 95 (2.47%) |  |
|  | 1,1,1,0,0,. | 48 (1.25%) |  |
|  | 1,1,1,0,.,. | 42 (1.09%) |  |
|  | 1,1,1,1,0,. | 18 (0.47%) |  |
| **Short Term (34.5%)** | 1,0,0,0,0,0 | 14824 (68.84%) | 1 |
|  | 1,1,0,0,0,0 | 4848 (22.51%) |  |
|  | 1,.,.,.,.,. | 479 (2.22%) |  |
|  | 1,0,0,.,.,. | 292 (1.36%) |  |
|  | 1,0,.,.,.,. | 282 (1.31%) |  |
|  | 1,0,0,0,0,. | 209 (0.97%) |  |
|  | 1,0,0,0,.,. | 195 (0.91%) |  |
|  | 1,1,.,.,.,. | 170 (0.79%) |  |
|  | 1,1,0,0,0,. | 82 (0.38%) |  |
|  | 1,1,0,.,.,. | 81 (0.38%) |  |
|  | 1,1,0,0,.,. | 72 (0.33%) |  |
| **Single  (37.3%)** | 0,0,0,0,0,0 | 20669 (88.87%) | 0 |
|  | 0,0,0,0,0,1 | 733 (3.15%) |  |
|  | 0,.,.,.,.,. | 457 (1.96%) |  |
|  | 0,0,.,.,.,. | 386 (1.66%) |  |
|  | 0,0,0,.,.,. | 361 (1.55%) |  |
|  | 0,0,0,0,0,. | 328 (1.41%) |  |
|  | 0,0,0,0,.,. | 323 (1.39%) |  |

Abbreviations: LCGA = Latent Class Growth Analysis; MH = Mental Health.

^a^ A value of 1 is used to denote that at least one fit note was issued in the given time interval, 0 denotes that no fit notes were issued, and a period indicates that the individual had missing data in the time interval.

^b^ Cell counts less than five are not shown in accordance with CPRD reporting guidelines (to reduce risk of patient identification).

The pattern is chronologically ordered in two monthly time intervals during the first year of follow-up since index fit note. For example, a pattern of 1,0,0,0,0,0 indicates that a fit note was issued in the first two months since index fit note, but not in the ensuing 10 months.

Note: trajectory prevalence is based on most likely latent class membership, not posterior probabilities, as individuals are treated as whole persons in the observed data, hence the posterior probabilities cannot be used.

**Table S.7.** Summary of Observed Fit Note Issuance Patterns During Year One Follow-Up for the ‘Intermittent Low’ Trajectory Class in the Optimal Five-Class LCGA Model for the Incident MSK Condition Fit Note Cohort

| **Outcome Pattern  (2 monthly)^a^** | **n (%)^b^** | **Median Intervals With Fit Note Received** |
| --- | --- | --- |
| 0,1,0,0,0,0 | 905 (12.44%) | 2 |
| 0,0,1,0,0,0 | 677 (9.31%) |  |
| 0,0,0,1,0,0 | 578 (7.95%) |  |
| 0,0,0,0,1,0 | 565 (7.77%) |  |
| 1,0,1,0,0,0 | 391 (5.38%) |  |
| 1,0,0,0,0,1 | 374 (5.14%) |  |
| 1,0,0,0,1,0 | 342 (4.70%) |  |
| 1,0,0,1,0,0 | 271 (3.73%) |  |
| 0,1,1,0,0,0 | 261 (3.59%) |  |
| 0,0,0,0,1,1 | 206 (2.83%) |  |
| 1,0,1,1,0,0 | 151 (2.08%) |  |
| 0,0,1,1,0,0 | 147 (2.02%) |  |
| 1,0,0,0,1,1 | 146 (2.01%) |  |
| 0,0,0,1,1,0 | 141 (1.94%) |  |
| 1,1,0,1,0,0 | 130 (1.79%) |  |
| 0,1,1,1,0,0 | 122 (1.68%) |  |
| 1,0,0,1,1,0 | 99 (1.36%) |  |
| 1,1,0,0,0,1 | 92 (1.27%) |  |
| 1,1,0,0,1,0 | 88 (1.21%) |  |
| 0,0,0,1,1,1 | 81 (1.11%) |  |
| 0,1,0,1,0,0 | 79 (1.09%) |  |
| 0,1,0,0,1,0 | 68 (0.94%) |  |
| 1,0,1,1,1,0 | 68 (0.94%) |  |
| 1,1,0,1,1,0 | 68 (0.94%) |  |
| 1,1,1,0,0,1 | 61 (0.84%) |  |
| 1,1,0,0,1,1 | 60 (0.83%) |  |
| 1,0,0,1,1,1 | 59 (0.81%) |  |
| 0,1,0,0,0,1 | 58 (0.8%) |  |
| 0,0,1,0,1,0 | 57 (0.78%) |  |
| 0,0,1,0,0,1 | 56 (0.77%) |  |
| 0,0,1,1,1,1 | 54 (0.74%) |  |
| 0,1,1,0,1,0 | 54 (0.74%) |  |
| 0,1,1,1,1,0 | 51 (0.70%) |  |
| 0,0,0,1,0,1 | 47 (0.65%) |  |
| 0,0,1,1,1,0 | 46 (0.63%) |  |
| 1,0,0,1,0,1 | 41 (0.56%) |  |
| 1,0,1,0,1,0 | 40 (0.55%) |  |
| 0,1,0,1,1,0 | 35 (0.48%) |  |
| 0,1,1,0,1,1 | 35 (0.48%) |  |
| 1,0,1,1,0,1 | 35 (0.48%) |  |
| 0,1,1,1,0,1 | 33 (0.45%) |  |
| 1,1,0,1,0,1 | 30 (0.41%) |  |
| 0,0,1,0,1,1 | 27 (0.37%) |  |
| 0,1,.,.,.,. | 27 (0.37%) |  |
| 1,0,1,0,1,1 | 27 (0.37%) |  |
| 0,1,0,1,1,1 | 25 (0.34%) |  |
| 1,0,1,0,0,1 | 25 (0.34%) |  |
| 0,1,0,0,1,1 | 19 (0.26%) |  |
| 0,1,0,.,.,. | 18 (0.25%) |  |
| 0,1,1,0,0,1 | 17 (0.23%) |  |
| 0,0,1,0,.,. | 12 (0.17%) |  |
| 0,0,1,1,0,1 | 12 (0.17%) |  |
| 0,1,0,0,.,. | 12 (0.17%) |  |
| 0,1,0,0,0,. | 12 (0.17%) |  |
| 0,0,0,0,1,. | 11 (0.15%) |  |
| 0,1,0,1,0,1 | 11 (0.15%) |  |
| 0,0,0,1,.,. | 10 (0.14%) |  |
| 0,1,1,.,.,. | 9 (0.12%) |  |
| 0,0,1,.,.,. | 8 (0.11%) |  |
| 1,0,1,.,.,. | 8 (0.11%) |  |
| 1,0,1,0,.,. | 8 (0.11%) |  |
| 0,0,0,1,0,. | 7 (0.10%) |  |
| 0,0,1,0,0,. | 7 (0.10%) |  |
| 0,1,1,1,.,. | 6 (0.08%) |  |
| 1,0,0,0,1,. | 6 (0.08%) |  |
| 1,0,0,1,.,. | 6 (0.08%) |  |

Abbreviations: LCGA = Latent Class Growth Analysis; MSK = Musculoskeletal.

^a^ A value of 1 is used to denote that at least one fit note was issued in the given time interval, 0 denotes that no fit notes were issued, and a period indicates that the individual had missing data in the time interval.

^b^ Cell counts less than five are not shown in accordance with CPRD reporting guidelines (to reduce risk of patient identification).

The pattern is chronologically ordered in two monthly time intervals during the first year of follow-up since index fit note. For example, a pattern of 1,0,0,0,0,0 indicates that a fit note was issued in the first two months since index fit note, but not in the ensuing 10 months.

Note: trajectory prevalence is based on most likely latent class membership, not posterior probabilities, as individuals are treated as whole persons in the observed data, hence the posterior probabilities cannot be used.

**Table S.8.** Summary of Observed Fit Note Issuance Patterns During Year One Follow-Up for the ‘Intermittent Low’ Trajectory Class in the Optimal Five-Class LCGA Model for the Incident MH Condition Fit Note Cohort

| **Outcome Pattern  (2 monthly)^a^** | **n (%)^b^** | **Median Intervals With Fit Note Received** |
| --- | --- | --- |
| 0,1,0,0,0,0 | 1295 (11.95%) | 2 |
| 1,0,1,0,0,0 | 682 (6.29%) |  |
| 0,0,1,0,0,0 | 662 (6.11%) |  |
| 1,0,0,0,0,1 | 655 (6.05%) |  |
| 0,0,0,1,0,0 | 621 (5.73%) |  |
| 0,0,0,0,1,0 | 598 (5.52%) |  |
| 0,1,1,0,0,0 | 476 (4.39%) |  |
| 1,0,0,1,0,0 | 439 (4.05%) |  |
| 1,0,0,0,1,0 | 415 (3.83%) |  |
| 1,1,0,1,0,0 | 302 (2.79%) |  |
| 1,0,1,1,0,0 | 260 (2.4%) |  |
| 0,0,0,0,1,1 | 259 (2.39%) |  |
| 1,1,0,0,0,1 | 246 (2.27%) |  |
| 1,0,0,0,1,1 | 231 (2.13%) |  |
| 0,1,1,1,0,0 | 204 (1.88%) |  |
| 0,0,0,1,1,0 | 196 (1.81%) |  |
| 0,0,1,1,0,0 | 185 (1.71%) |  |
| 1,0,0,1,1,0 | 184 (1.7%) |  |
| 1,1,0,0,1,0 | 165 (1.52%) |  |
| 1,1,0,1,1,0 | 146 (1.35%) |  |
| 1,1,1,0,0,1 | 141 (1.3%) |  |
| 1,0,1,1,1,0 | 136 (1.26%) |  |
| 1,0,0,1,1,1 | 123 (1.14%) |  |
| 1,1,0,0,1,1 | 118 (1.09%) |  |
| 0,1,0,1,0,0 | 115 (1.06%) |  |
| 0,1,1,1,1,0 | 110 (1.02%) |  |
| 0,0,0,1,1,1 | 106 (0.98%) |  |
| 0,0,1,1,1,0 | 89 (0.82%) |  |
| 0,1,0,0,0,1 | 87 (0.8%) |  |
| 0,0,0,1,0,1 | 81 (0.75%) |  |
| 0,1,1,0,1,0 | 72 (0.66%) |  |
| 0,0,1,0,1,0 | 69 (0.64%) |  |
| 0,0,1,1,1,1 | 69 (0.64%) |  |
| 1,0,1,0,1,0 | 69 (0.64%) |  |
| 0,1,0,1,1,0 | 67 (0.62%) |  |
| 0,1,1,0,1,1 | 66 (0.61%) |  |
| 0,1,0,0,1,0 | 64 (0.59%) |  |
| 1,0,1,1,0,1 | 62 (0.57%) |  |
| 0,0,1,0,0,1 | 58 (0.54%) |  |
| 0,1,0,1,1,1 | 56 (0.52%) |  |
| 1,0,1,0,0,1 | 55 (0.51%) |  |
| 1,1,0,1,0,1 | 53 (0.49%) |  |
| 1,0,1,0,1,1 | 51 (0.47%) |  |
| 1,0,0,1,0,1 | 50 (0.46%) |  |
| 0,1,0,0,1,1 | 47 (0.43%) |  |
| 0,1,.,.,.,. | 45 (0.42%) |  |
| 0,1,1,1,0,1 | 42 (0.39%) |  |
| 0,1,1,0,0,1 | 39 (0.36%) |  |
| 0,0,1,0,1,1 | 35 (0.32%) |  |
| 0,1,0,1,0,1 | 33 (0.3%) |  |
| 0,1,0,.,.,. | 30 (0.28%) |  |
| 0,0,1,1,0,1 | 28 (0.26%) |  |
| 0,1,0,0,.,. | 28 (0.26%) |  |
| 0,0,1,.,.,. | 25 (0.23%) |  |
| 1,0,1,.,.,. | 25 (0.23%) |  |
| 0,0,0,1,.,. | 20 (0.18%) |  |
| 0,1,0,0,0,. | 20 (0.18%) |  |
| 0,1,1,.,.,. | 20 (0.18%) |  |
| 1,0,1,0,.,. | 19 (0.18%) |  |
| 1,0,1,0,0,. | 13 (0.12%) |  |
| 0,0,0,0,1,. | 12 (0.11%) |  |
| 0,1,1,1,.,. | 12 (0.11%) |  |
| 1,0,0,0,1,. | 12 (0.11%) |  |
| 1,0,0,1,.,. | 12 (0.11%) |  |
| 1,0,1,1,.,. | 12 (0.11%) |  |
| 0,0,1,0,0,. | 11 (0.1%) |  |
| 0,1,1,0,.,. | 11 (0.1%) |  |
| 1,0,0,1,0,. | 11 (0.1%) |  |
| 0,0,1,0,.,. | 10 (0.09%) |  |
| 0,0,0,1,0,. | 9 (0.08%) |  |
| 0,1,1,0,0,. | 9 (0.08%) |  |
| 0,0,1,1,.,. | 8 (0.07%) |  |
| 1,1,0,1,.,. | 7 (0.06%) |  |
| 1,0,1,1,0,. | 6 (0.06%) |  |

Abbreviations: LCGA = Latent Class Growth Analysis; MH = Mental Health.

^a^ A value of 1 is used to denote that at least one fit note was issued in the given time interval, 0 denotes that no fit notes were issued, and a period indicates that the individual had missing data in the time interval.

^b^ Cell counts less than five are not shown in accordance with CPRD reporting guidelines (to reduce risk of patient identification).

The pattern is chronologically ordered in two monthly time intervals during the first year of follow-up since index fit note. For example, a pattern of 1,0,0,0,0,0 indicates that a fit note was issued in the first two months since index fit note, but not in the ensuing 10 months.

Note: trajectory prevalence is based on most likely latent class membership, not posterior probabilities, as individuals are treated as whole persons in the observed data, hence the posterior probabilities cannot be used.

# S5: Exploration of Growth Mixture Modelling for Optimal MSK Condition Model

**Figure S.1.** Five-Class Models for the Cohort with Index Fit Note Due to a MSK Condition, Based on Interval Approach 2 (Two Monthly Intervals, Years One to Three Data), Based on LCGA (Left) and GMM-CI (Right)

Abbreviations: LCGA = Latent Class Growth Analysis; GMM-CI = Growth Mixture Modelling, using a Class Invariant approach; MSK = Musculoskeletal.
For each trajectory, the solid lines represent the probability of fit note issuance in the given time interval (model estimated data), whilst the dotted lines represent the proportion of individuals issued a fit note (observed data). Trajectory class prevalence is shown, derived from a count based on posterior probability
